# Synthesis of evidence on the use of ecological momentary assessments to monitor health outcomes after traumatic injury: rapid systematic review

**DOI:** 10.1186/s12874-022-01586-w

**Published:** 2022-04-22

**Authors:** Rebecca J. Mitchell, Rory Goggins, Reidar P. Lystad

**Affiliations:** Australian Institute of Health Innovation, Macquarie University, Level 6, 75 Talavera Road, Sydney, NSW 2109 Australia

**Keywords:** Ecological momentary assessment, Experience sampling, Injury, Health outcome

## Abstract

**Background:**

With the increasing use of mobile technology, ecological momentary assessments (EMAs) may enable routine monitoring of patient health outcomes and patient experiences of care by health agencies. This rapid review aims to synthesise the evidence on the use of EMAs to monitor health outcomes after traumatic unintentional injury.

**Method:**

A rapid systematic review of nine databases (MEDLINE, Web of Science, Embase, CINAHL, Academic Search Premier, PsychINFO, Psychology and Behavioural Sciences Collection, Scopus, SportDiscus) for English-language articles from January 2010–September 2021 was conducted. Abstracts and full-text were screened by two reviewers and each article critically appraised. Key information was extracted by population characteristics, age and sample size, follow-up time period(s), type of EMA tools, physical health or pain outcome(s), psychological health outcome(s), general health or social outcome(s), and facilitators or barriers of EMA methods. Narrative synthesis was undertaken to identify key EMA facilitator and barrier themes.

**Results:**

There were 29 articles using data from 25 unique studies. Almost all (84.0%) were prospective cohort studies and 11 (44.0%) were EMA feasibility trials with an injured cohort. Traumatic and acquired brain injuries and concussion (64.0%) were the most common injuries examined. The most common EMA type was interval (40.0%). There were 10 key facilitator themes (e.g. feasibility, ecological validity, compliance) and 10 key barrier themes (e.g. complex technology, response consistency, ability to capture a participant’s full experience, compliance decline) identified in studies using EMA to examine health outcomes post-injury.

**Conclusions:**

This review highlighted the usefulness of EMA to capture ecologically valid participant responses of their experiences post-injury. EMAs have the potential to assist in routine follow-up of the health outcomes of patients post-injury and their use should be further explored.

**Supplementary Information:**

The online version contains supplementary material available at 10.1186/s12874-022-01586-w.

## Background

The growing recognition by health service agencies of their need to demonstrate provision of value-based care, has resulted in a shift in metrics used to monitor healthcare performance [1]. Foremost in benchmarking performance among health facilities is the monitoring and reporting of health outcomes in clinical populations in-hospital and post-discharge [2–4]. With the increasing use of mobile technology, one technique that may enable routine monitoring of patient health outcomes and patient experiences of care is ecological momentary assessments (EMAs).

EMAs, experiencing sampling methods or ambulatory assessments allow snap-shots into real-life moments by enabling self-collection of thoughts, behaviours, symptoms, activities, experiences, or biometric data (e.g. heart rate), in real-time from a defined population [5, 6]. Information is usually collected for short, specific periods either after a specific event or experience (i.e. event-contingent sampling), at fixed, regular intervals throughout a day (i.e. interval-contingent sampling) or at random time points during a day (i.e. signal-contingent sampling) [7]. These repeated measurements are being collected increasingly via mobile devices, including smart phones or sensor equipment.

The information collected using EMAs is usually recorded in a natural environmental situation and allows temporal sequences of symptoms or conditions to be monitored and relationships, and often interdependencies, between conditions to be explored [5, 8, 9]. The use of EMAs can improve data validity by reducing many data collection biases, such as retrospective recall bias, and data entry or transcription errors [5, 8]. EMAs also allows for the timely acquisition of information regarding an individual’s health outcomes and, when needed, swiftly acted upon [9].

EMAs have been frequently used to monitor risk factors and behaviours for intentional injuries, including suicidal thoughts, behaviours, and acts of self-harm [10], but their use has been fairly limited in monitoring health outcomes after other types of injuries. Around 973 million individuals sustain a traumatic injury (such as fractures, dislocations, open wounds, sprains or strains) worldwide each year that required some form of healthcare [11]. A serious injury can have an adverse impact on the individual, their family, and local community [12].

Many seriously injured individuals can experience ongoing mobility and functional limitations, depression, anxiety and post-traumatic stress disorder (PTSD) [13]. Therefore, the ability to monitor physical, psychological, and social health outcomes after injury, along with experiences of service use, and social participation would be advantageous to identify the need for interventions, ongoing service needs, and service planning, including use of primary care and allied health. The aim of this rapid review is to synthesise the evidence on the use of EMAs to monitor health outcomes after traumatic unintentional injury.

## Method

This rapid review synthesises the evidence on the use of EMAs to monitor different types of health outcomes after sustaining an injury. The review examines information on the type of EMA tools used, follow-up periods, the different tools and methods used to monitor health outcomes, and the facilitators and barriers identified to using EMAs to monitor health outcomes post-injury. This review adhered to the Preferred Reporting Items for Systematic Review and Meta-analyses (PRISMA) statement [14].

### Definitions

Research studies were included in the rapid review if they used EMAs to monitor the health outcomes of an individual after sustaining a traumatic injury. An EMA is a method by which information is captured at multiple time periods regarding an individuals’ current health (e.g. physical or psychological) state or experiences in real-time [5]. A traumatic injury was considered to be “a bodily lesion at the organic level caused by acute exposure to physical agents such as mechanical energy, heat, electricity, chemicals, and ionizing radiation interacting with the body in amounts or at rates that exceed the threshold of human tolerance. In some cases, injuries result from the sudden lack of essential agents such as oxygen or heat” [15]. There were no restrictions on the type of injury sustained. However, intentional injuries following self-harm or interpersonal violence were excluded from the review as EMAs have been frequently used to monitor risk factors and behaviours for intentional injuries, whereas use of EMAs has been limited to monitor outcomes after unintentional injuries.

Individual health outcomes could either capture health states in the short-, medium- or long-term. Health outcomes could include information on physical health or pain outcomes (e.g. physical functioning, mobility, activities of daily living (ADLs), pain, medication use), psychological health (e.g. depression, anxiety, stress, PTSD), or general health and social outcomes (such as quality of life (QoL), social activity participation, biometric data e.g. heart rate, hours spent sleeping, step count).

### Data sources and eligibility criteria

A systematic search was conducted using nine databases: MEDLINE, Web of Science, Embase, CINAHL, Academic Search Premier, PsychINFO, Psychology and Behavioural Sciences Collection, Scopus, SportDiscus. The search strategy was developed with a university librarian and included the following search terms: (ecological momentary assessment* OR Ecological Momentary Assessment OR momentary assessment* OR EMA OR experience sampling OR ambulatory assessment* OR event sampling), AND (injur* OR accident OR trauma* OR accident* OR wound* OR lesion* OR bruise* OR abrasion* OR harm) (see Additional file 1 for full search strategy).

Studies were excluded if the article was a systematic or other type of review, a single case report, a study protocol, or if there was insufficient detail regarding the health outcome(s) examined. Results were limited to English-language articles that were published in peer-review journals from 1 January 2010 to 21 September 2021. Snowballing of reference lists from the articles was conducted to identify any potential articles not previously identified.

### Abstract screening

The title, abstract and citation information relating to each study identified during the database searches was imported to Endnote X20 and duplicates removed. The abstracts were independently assessed for inclusion by three reviewers (RG, RL, RM), who met regularly to discuss any uncertainties. If the abstract did not report on how EMAs were used to monitor health outcomes after unintentional injury it was excluded. Any disagreements on abstract inclusion were discussed and consensus achieved. Abstract screening was independently verified for accuracy by dual screening each abstract in pairs, with 99.0% percent agreement achieved during the initial abstract screen (i.e. RG/RL and RG/RM). After discussion, consensus was obtained to include *n* = 34 abstracts to the full-text screening stage.

### Full-text screening, data extraction and quality review

In the full-text screening each study was assessed by three reviewers (RG, RL, RM). Any study that did not meet the inclusion criteria was excluded. For studies that met the inclusion criteria, key characteristics of each study were extracted during the full-text review by one reviewer (RG), including: authors and publication year; review objective/aim; study type, country and data collection timeframe, population characteristics, age and sample size, EMA type (i.e. signal, event or interval) and follow-up time period(s), EMA assessment tool(s), physical health or pain outcome(s), psychological health outcome(s), general health or social outcome(s), and facilitators or barriers of EMA methods identified during the study by the authors. Data extraction results were independently verified for accuracy by a second reviewer (RM) and any disagreements were discussed. The methodological quality of the articles was assessed by one reviewer (RG) using the CASP Cohort checklist [16] or the CASP RCT checklist [17]. Any clarifications regarding methodological quality were discussed between all reviewers.

### Narrative synthesis

The information on the included studies in the data extraction table was compared and a narrative synthesis was undertaken of the facilitators and barriers by one reviewer (RM) and these were appraised by a second reviewer (RL). The narrative synthesis involved reading and reviewing each of the facilitators and barriers identified in the discussion section of each article. Then an inductive, iterative process was used to categorise each factor identified as either as a facilitator or a barrier based on the key factor theme (e.g. a facilitator of ‘data collection minimises recall bias’ was categorised as ‘reliability’ and a barrier of ‘EMA is potentially a time burden for study participants’ was categorised as ‘time-burden’). Each factor was allocated to one theme, but several facilitators or barriers could be identified in each article.

## Results

**Fig. 1:**
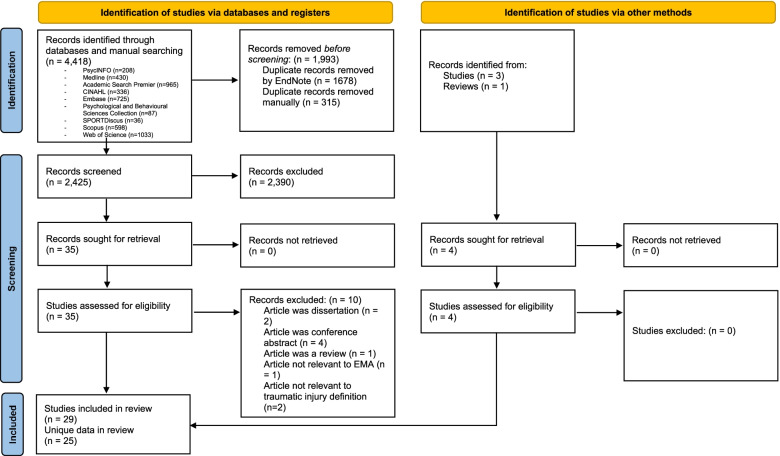
PRISMA flow diagram^1^ ^1^PRISMA Flow diagram attribution: Page M et al. The PRISMA 2020 statement: an updated guideline for reporting systematic reviews. BMJ 2021;372:n71. doi: 10.1136/bmj.n71

There were 4418 records identified during the database searches. After removing duplicates, 2425 records remained. After abstract screening, 35 full-text records were assessed for eligibility, along with four records identified after snowballing. A final 29 articles using data from 25 unique studies were included in the rapid review (Fig. 1).

Almost all (84.0%) unique studies were prospective cohort studies, with four (16.0%) randomised control trial (RCTs) designs. Eleven (44.0%) studies were feasibility trials of the effectiveness of using EMA to record information with a traumatically injured cohort. The majority of studies were conducted in the United States (84.0%), with one (4.0%) study each in Canada, Germany, the Netherlands, and Spain. Traumatic brain injury (TBI) (i.e. *n* = 5 mild TBI, *n* = 2 severe TBI, and *n* = 2 TBI – severity not specified), concussion (*n* = 5), and acquired brain injury (*n* = 2) were the most common injuries examined (64.0%). Traumatic injury, such as fractures open wounds or anterior cruciate ligament injuries (20.0%; *n* = 5) and spinal cord injury (16.0%; *n* = 4) were also examined. The most common EMA type was interval (40.0%; *n* = 10), with nine (36.0%) random, and three (12.0%) event types. One (4.0%) study used both interval and event EMAs, and one study (4.0%) used all three EMA types. A variety of assessment measures were used to record information on physical or pain, psychological, general health and social outcomes post-injury using EMA. Only nine (36.0%) studies recorded information on social activity participation. Almost all (89.7%) authors identified at least one facilitator or barrier of using EMA (Table 1).

**Table 1:** Characteristics of studies using ecological momentary assessments to monitor health outcomes after injury

Authors and publication year	Objective/aim	Study type	Country (data collection dates)	Injured population, age and sample size	EMA type & follow-up time period(s)	EMA assessment tool(s)	Physical health or pain outcome(s)	Psychological health outcome(s)	General health or social outcome(s)	Facilitators or barriers of EMA methods identified by study authors
**Traumatic brain injury, concussion, and acquired brain injury**										
Albanese et al. 2021 [18]	Test whether distress intolerance predicted traumatic intrusions following a trauma film.	Prospective cohort study.	Florida, United States (unspecified date)	*n* = 70 aged ≥18 years with mild traumatic brain injury (mTBI) or anxiety sensitivity.	Interval. 1. 3 h after film; 2. 10 am, 3 pm, & 8 pm each day for 13 days after film.	1. Text messages with survey link to Qualtrics online survey.	N/A	1.Natualistic trauma intrusions. 2.Depression Anxiety & Stress Scale (DASS-21).	N/A	N/A
Betthauser et al. 2021 [19]	Assessed the feasibility of design elements of a yoga-based interventional trial for post-concussive headaches among Veterans with mTBI, as well as the acceptability of the intervention.	Randomised controlled acceptability and feasibility trial.	United States (2017–2019)	*n* = 27 to 54 Veterans aged ≥18 years with mTBI at various intervention stages.	Event. 1. Study follow-up visits at time 3, 4 & 5.	1. Either text message, web-based or pen & paper.	1. Brief Pain Inventory. 2. Headache Impact Test (HIT-6) impact of headaches on activities of daily living (ADL). 3. K Scale: Survey of Headache Impact (KS-SHI). 4. Short-Form McGill Pain Questionnaire (SF-MPQ). 5. Medication taken to treat headaches.	1. Neuro-behavioural Symptom Inventory (NSI). 2. Perceived Stress Scale (PSS).	1. Patient-Reported Outcomes Measurement Information System (PROMIS). 2. Short-Form Health Survey (SF-36). 3. Barriers & facilitators to activity participation. 4. Home yoga practice.	**Facilitators:** 1. Feasibility of web-based ecological momentary assessment (EMA) demonstrated. 2. Study results support the use of EMA procedures to track an intervention. 3. A modification to EMA facilitated the study team to contact and encourage participants. **Barriers** N/A
Forster et al. 2020 [20]	To establish the feasibility of using EMA (i.e., in terms of patients’ compliance) in patients with an acquired brain injury (ABI). To map fluctuations in patients’ responses. To determine whether patients’ compliance and their fluctuations in response behaviour were related to their level of functioning.	Prospective cohort pilot study	Germany (unspecified date)	*n* = 15 individuals with an ABI with cognitive and motor impairments. Aged 18–70 years.	Random. 8 prompts per day for 7 days. Prompts were given between 8 am and 8 pm with at least an hour between prompts.	1. The software movisensXS, App version 1.3.0 via Android smartphones (Motorola Moto G, third generation)	N/A	1. Rasch-based Depression Screening using a five-point Likert scale. 2. Attention Network Test to assess attention and reaction time computer-based reaction time. 3. The Verbal Learning and Memory Test to measure declarative verbal memory, learning performance, long-term encoding, and recognition performance.	1. Ability to conduct 10 basic daily functions using the Barthel Index.	**Facilitators** 1. Patients’ compliance did not differ between weekdays and weekend. 2. Patients able to assess and monitor their own symptoms. 3. Ability for results to be shared with the treating clinician. 4. Sustains ecologically valid results. **Barriers** 1. Compliance decreased with every passing testing day. 2. The higher frequency of EMA prompts correlated with lower compliance as burden increased. 3. Difficult to gauge how reliable and realistic the patients answers were. 4. Participant mood and emotion may have been influenced by high frequency of prompts. 5. The use of EMA might also be limited by other psychological factors such as social desirability and patients’ individual differences.
Hart et al. 2019 [21]	To describe and provide the rationale for a randomized controlled trial for depression or anxiety after moderate to severe TBI, which will test two treatments based on behavioural activation (BA).	Randomised controlled trial	United States (unspecified date)	*n* = 60 individuals with TBI that was sustained at least 6 months prior to enrolment. Consciousness must be altered and depression and anxiety present. Participants aged≥18 years.	Random. 5 times per day within a 14-h window for 8 weeks.	1. Collected via the LifeData System via RealLife Exp (a mobile app).	N/A	1. Emotional status/ behaviour - Global Severity Index of the Brief Symptom Inventory (BSI) -18, Environmental Reward Observation Scale (EROS), the Behavioral Activation for Depression Scale (BADS).	1. Participant Assessment with Recombined Tools-Objective (PART-O) to assess societal/ community participation. 2. Satisfaction with life Scale (SWLS). 3. Quality of life after brain injury (QOLIBRI). 4. Patient Global Impression of Change. 5. Cognitive/ functional status using Wechsler Abbreviated Scale of Intelligence. 6. Functional status - Extended Glasgow Outcome Scale.	**Facilitators** 1. The incorporation of commonly used technology in the form of smartphone apps has the potential to enhance the delivery of treatment in this population. **Barriers** N/A
Juengst et al. 2015a [22] Juengst et al. 2019b [23]	A) Pilot feasibility and validity of a mobile health (mHealth) system for tracking mood-related symptoms after TBI. B) To investigate within-person variability in daily self-reported emotional and fatigue symptoms and factors associated with high within person variability among individuals with chronic TBI.	A) Prospective cohort pilot study. B) Secondary analysis of prospective cohort pilot study	United States (unspecified date)	A) *n* = 20 individuals with TBI and aged ≥18 years. B) *n* = 18 adults with chronic TBI (2–27 years post-injury) who owned and could independently use an Apple or Android device. Participants aged 22–60 years.	Interval. Daily assessments for 8 weeks.	1. Conducted via mHealth system delivered through iPerform application.	N/A	1. Depressive symptoms using the Patient Health Questionnaire 2 (PHQ2). 2. Generalised Anxiety Disorders (GAD2). 3. Positive and Negative Affect Scale (PANAS). 4. Patient Health Questionnaire 9 (PHQ9). 5. Generalised Anxiety Disorders 7 (GAD7).	1. Impact of fatigue on daily life using a 7-point Likert scale	A) **Facilitators** 1. Participants showed good compliance and high satisfaction with the method of EMA. 2. Ecological valid results were demonstrated. 3. The simple interface on the app proved to be helpful with participants with TBI and could successfully complete assessments. 4. A schedule feature resulted in great compliance and satisfaction. **Barriers** 1. Some participants frequently completed the wrong assessment. 2. Some participants completed too many assessments per day. B) **Facilitators** 1. Feasibility of mobile phone-based EMA demonstrated. 2. Use of EMA may reduce misidentification of individuals with clinically significant symptoms. 3. Higher frequency repeated symptom assessment in a natural environment over a short period could provide a more valid measure of emotional symptoms and a better indicator of clinically meaningful change at the individual level. **Barriers** 1. Single daily assessments may not be a valid representation of symptoms/ progression. 2. Method of self-reporting may contribute to variability.
Lenaert et al. 2019 [24]	1. To investigate the feasibility of using the experiencing sampling method (ESM) in individuals with ABI. 2. To explore the usability of ESM data on a clinical level by illustrating the interactions between person, environment, and affect.	Prospective cohort study.	The Netherlands (July 2014–March 2015)	*n* = 17 individuals with ABI aged 18–65 years.	Random. 10 semi-random beeps throughout the day between 7.30 am and 10.30 pm for 6 days.	1. PsyMate (smart eHealth GmbH, Luxembourg) - a small electronic device with a touch-screen interface.	1. Physical well-being using a 7-point Likert scale.	1. PANAS. 2. Mood and self-esteem using a 7-point Likert scale.	1. Context and activities related questions using a multi-choice format. 2. Appraisal of context and activities using a bipolar scale ranging from −3 to + 3.	**Facilitators** 1. Data collection method had little interference on daily activities despite high volume of beeps. 2. User-friendly interface allowed for easy completion of assessments. 3. Familiarity with the questions over time reduced time and energy needed to answer them. 4. Data collection method had little influence on mood and feelings promoting ecological validity. 5. Users were able to monitor own progress and rehabilitation. **Barriers** N/A
Pavliscsak et al. 2016 [25]	1. To examine engagement with a mobile application (i.e. mCare) for wounded Veterans rehabilitating in their communities. 2. To examine associations between Veterans’ background characteristics and their engagement with mCare.	Prospective Randomised Controlled Trial	United States (Unspecified specified)	*n* = 95 participants who received mCare. This included individuals with TBI (no distinction was made between those with TBI and those with other health issues). Participants were aged ≥18 years.	Interval. Daily assessment (unspecified time) for 36 weeks.	1. EMA delivered via the mCare mobile application. 2. Text messages to notify of new information on the mCare application.	N/A	N/A	1. General participation.	**Facilitator** 1. Findings suggest mCare can and will be adopted by Veterans in a community setting, even those with cognitive and emotional difficulties. **Barriers** 1. Participants’ exposure to mCare declined systematically as Veterans out processed from rehabilitation.
Rabinowitz et al. 2020 [26]	To illustrate a novel framework for conceptualizing, collecting, and analysing concussion symptom data.	Prospective cohort pilot study	United States (Unspecified date).	*n* = 10 recently concussed adolescents and young adults aged 15–35 years.	Interval. Five assessments per day for 20 days. (morning, early afternoon, late afternoon, evening, and night)	1. Collected via the LifeData System (via RealLife Exp (a mobile app)).	N/A	N/A	1. 22-item Post-concussion Symptom Scale (PCSS).	N/A
Rabinowitz et al. 2021 [27]	To describe where, with whom, and how time was spent daily, and to characterise positive and negative affect, boredom, enjoyment, and perceived accomplishment as a function of time, activity, location, and social context, in people with chronic moderate-severe TBI and depression/anxiety.	Prospective cohort study.	United States (5 April 2018–1 Feb 2020)	*n* = 23 individuals with TBI and at least mild depression or anxiety. Mean age 47.7 years.	Random. Notified 5 times in 14-h window for ~ 2 weeks.	1. Collected via the LifeData System (via RealLife Exp (a mobile app)).	N/A	1. Positive and Negative Affect using 20-item PANAS.	1. General well-being and activity using multi-choice. 2. Free-text responses were able to be provided for each question to qualify responses. 3. Activities in last hour recorded, including who primarily doing activity with.	**Facilitators** N/A **Barriers** 1. Closer monitoring with more proactive troubleshooting could have improved participants’ response rates.
Smith et al. 2012 [28]	To assess the utility of mHealth technologies, including personal digital assistant-based EMA and two-way interactive text (SMS) messaging, for providing treatment feedback to clinicians, encouraging and motivating Veterans throughout treatment, and monitoring participants for relapse after treatment discharge.	Prospective cohort pilot study	United States (Unspecified date).	*n* = 27 male veterans suffering from PTSD and/or mTBI. Distinctions between those with PTSD from those with mTBI were not made. Age unspecified.	Interval, Random and Event. Active phase: 1. Daily assessment. Was initially randomised then at routine intervals. Follow-up phase 3 months post-discharge: 1. Daily at either 9 am or 5 pm.	1. Electronic survey tool designed to support data collection via PDAs was used in active phase. 2. Assessments sent and completed via SMS (provided by LifeWIRE corporation) in follow-up phase	1. Level of pain using a 1–10 Likert scale. 2. Symptoms Checklist-6.	1. Depressive symptoms using BriefCOPE, and Beck Depression Inventory-II.	1. Types of hassles and uplifts. 2. General activities.	**Facilitators** 1. After data collection was changed from random to routine intervals, response rate improved. **Barriers** 1. EMA at random times during waking hours was disruptive to participants’ schedules and led to low response rate. 2. Participants were resistant to carrying two electronic devices and routinely left their assigned PDA in the housing unit during the day, which contributed to the low EMA response rate. 3. The PDAs were viewed as being clunky and out of date compared with smart phones. 4. Participants indicated that they did not believe the data they were providing were doing any good because they could not see any effect on their treatment. On a few occasions participants temporarily ceased participating in EMA data collection after researchers and clinicians failed to respond to a stress or crisis event recorded in EMA data.
Suffoletto et al. 2013 [29]	To examine whether patients with mTBI receiving text message-based education and behavioural support had fewer and less severe post-concussive symptoms than those not receiving text messaging support, and determine the feasibility of using text messaging to assess daily symptoms and provide support to patients with mTBI.	RCT pilot	United States, (July–September 2012	*n* = 43 adults aged ≥18 years with mTBI. *n* = 18 in intervention group and *n* = 25 in control group.	Interval. Three daily questions at 9 am, 1 pm and 5 pm over 14 days	SMS messages.	1. Pain scale via have you had a headache in the last 24 h rating using 4-item Likert scale.	1. Difficulty concentrating in last 24 h rated using 4-item Likert scale. 2. Irritability or anxiety in last 24 h rated using 4-item Likert scale.	N/A	**Facilitators** 1. Provision of self-care support messages focused on symptom-specific education, reassurance, and management guidance lowered irritability or anxiety during the acute recovery period. **Barriers** N/A
Sufrinko et al. 2019 [30]	To evaluate mobile EMA as an approach to measure sport-related concussion symptoms, explore the relationships between clinical outcomes and mobile EMA, and determine whether mobile EMA was advantageous for predicting recovery outcomes compared to traditional symptom report.	Prospective cohort study.	United States (September 2016 – December 2016)	*n* = 20 athletes aged 12–19 years with SRC.	Random. Pseudo-randomised in 3 time blocks (i.e. Mon-Fri 7-8 am, 3-4 pm and 9-10 pm and Sat-Sun 9-10 am, 3-4 pm and 9-10 pm) daily for 4 weeks and clinical assessment at visit 1 and visit 2.	1. EMA surveys were administered via mobile EMA mobile application (Ilumivu, Inc). 2. The application utilized push notifications prompting the participant to open the application.	1. Vestibular/ Ocular Motor Screening rated using 0–10 Likert-scale assessing intensity for 4 symptoms (i.e. headache, dizziness, nausea, and fogginess).	1. Neurocognitive using the Immediate Post-Concussion Assessment and Cognitive Testing (ImPACT).	1. 22-item Post-concussion Symptom Scale (PCSS) rated using 7-point Likert scale.	**Facilitators** 1. Mobile EMA better predicted recovery time than Post-Concussion Symptom Scale (PCSS). 2. Mobile EMA data across recovery better predicted recovery duration compared with PCSS score at any clinic visit, but illustrated symptom patterns that may further inform clinical profiles and guide treatment recommendations. **Barriers** 1. Participants were less likely to respond as days since injury increased. 2. Results reflected a diminishing response rate throughout the course of the study. May be due to: a. Mobile EMA data collected for a longer duration than previous studies. b. Intervals were less frequent than other studies that yielded higher compliance with more intervals. c. Participants’ symptoms resolving and disinterest in completing mobile EMA.
Trbovic et al. 2021 [31]	To use actigraphy and mobile EMA to examine the relationship between sleep parameters and next day symptoms.	Prospective cohort study.	United States (September 2016 – December 2016)	*n* = 17 athletes aged 12–19 years with recent concussion.	Interval. Thee scheduled assessments per day within 1 h of prompt (i.e. morning – 7 am school days and 9 am weekends, afternoon – 3 pm, evening – 9 pm). By fourth week around half the participants had recovered, and sample size was underpowered beyond 3 weeks post-injury.	1. EMA surveys were administered via mobile EMA mobile application (Ilumivu, Inc). 2. The application utilized push notifications prompting the participant to open the application. 3. Participants wore an Actigraph GT3x + on their nondominant wrist for 24 h per day.	1. Somatic symptoms using PCSS and 7-point Likert scale.	1. Affective symptoms using PCSS and 7-point Likert scale.	1. PCSS to capture symptom intensity, including sleep-related symptoms. 2. Sleep and activity were measured by the Actigraph GT3x + . 3. Sleep-related symptoms rated using 7-point Likert scale.	N/A
Wiebe et al. 2016 [32]	To determine the feasibility of EMA following youth concussion, gather real-time reports of cognitive and physical activity, and compare objective measures with real-time reported symptoms among youths during recovery after concussion.	Prospective cohort pilot study	United States. (Unspecified date).	*n* = 34 recently concussed adolescents aged 11–19 years.	Random. Several random prompts daily for approximately 2 weeks after their initial office visit.	1. Participants wore an accelerometer2. iPod Touch (Apple) loaded with an app that gave random prompts	N/A	1. Daily cognitive rest and exertion were measured as number of text messages sent, minutes of screen time and gaming and minutes of reading or school work.	1. PCSS. 2. Activity questionnaire	**Facilitators** 1. Ecological validity was shown. **Barriers** N/A
Worthen-Chaudhari et al. 2017 [33]	To evaluate whether a mobile health application that employs elements of social game design could compliment medical care for unresolved concussion symptoms.	Prospective cohort study.	United States (Phase I – 13 Aug 2014–7 Jan 2015; Phase II – 7 Jan 2015–4 Nov 2015)	Phase I *n* = 20; Phase II *n* = 19 adolescents aged 13–18 years, with concussion symptoms ≥3 weeks post-injury.	Event. Participants asked to log activity at the frequency of one logged activity per day for 5 days each week, for a target of 15 logged activities over the first 3 weeks between pre-and post-test.	1. EMA conducted via a mobile application - SuperBetter.	1. Depression, measured by the Center for Epidemiological Studies–Depression Child (CES-DC)	1. Optimism, measured by the Life Orientation Test-Revised (LOT-R)	1. Severity of 22 Concussion symptoms using Sports Concussion Assessment Tool-3 (SCAT-3) checklist.	**Facilitators** 1. Youth were able to use the SuperBetter app in conjunction with traditional medical care for post-concussive symptoms and were satisfied with use of the app. 2. Participants who used the app to complement medical care had more relief from concussion symptoms than those who had traditional medical care alone. 3. The gameful and/or social interactive design of SuperBetter was effective to improve optimism. 4. Patients able to assess and monitor their own symptoms. **Barriers** N/A
**Spinal cord injury**										
Carlozzi et al. 2018 [34]	Investigated the most efficient means of measuring pain intensity and pain interference comparing EMA to end of day (EOD) data, with the highest level of measurement reliability as examined in individuals with spinal cord injury (SCI).	Prospective observational study.	United States (unspecified date)	*n* = 131 individuals with SCI aged ≥18 years. Participants also required to endorse ≥4 out of 10 average pain.	Interval. 1. Five times throughout the day (upon waking, 11 am, 3 pm, 7 pm, and bedtime)	1. Wrist-worn accelerometer enhanced with a user interface for entry of self-report data (i.e. the PRO-Diary; CamNTech, Cambridge, UK).	1. Pain 5-point Likert scale. 2. PROMIS pain intensity. 3. SCI-QOL pain interference. 4. 10-item short form pain interference 5. Pain interference 5-point Likert scale.	N/A	N/A	**Facilitators:** 1. EMA is easy to complete as responses are less reliant on memory. 2. The timing of EMA assessments does not impact reliability. **Barriers:** 1. EMA requires more sophisticated analytical hardware for monitoring and data capture. 2. EMA is potentially a time burden for study participants. 3. The presence of a floor effect for EMA pain interference presented an analytical challenge in our data. 4. Calibration data may over- or under-estimate the pain ratings of participants in this study. 5. Pain in SCI is multifaceted, and thus ratings of pain intensity do not capture the full breadth of the pain experience in individuals with SCI.
Carlozzi et al. 2021 [35]	Examined the effect of sleep quality on same day Health Related Quality of Life (HRQoL).	Prospective cohort study.	United States (unspecified date)	*n* = 170 individuals with SCI aged ≥18 years	Interval. 1. Three times a day (i.e. morning, afternoon, evening) for 7 days.	1. Smart phone or paper diary. 2. E4 wristband (Empatica) recorded heart rate variability, electrodermal activity, body movement (accelerometer data), & skin temperature.	1. Pain 10-point Likert scale. 2. PROMIS pain intensity. 3. PROMIS pain interference.	1. Thinking 10-point Likert scale. 2. PROMIS cognitive function abilities. 3. PROMIS depression. 4. PROMIS anxiety.	1. Fatigue 10-point Likert scale. 2. PROMIS sleep disturbances. 3. PROMIS sleep-related impairment. 4. PROMIS fatigue. 5. PROMIS ability to participate in social roles and activities.	**Facilitators:** 1. Does not take very long for participants to habituate to a monitoring device (i.e. E4). 2. Ability to examine temporal relationships among symptoms. 3. Data collection minimises recall bias. 4. Maximises ecological validity of responses. **Barriers:** 1. The E4 wristband does not have established algorithms for evaluating sleep in the general population or in individuals with SCI. 2. Self-reported sleep quality and objective sleep measured by E4 was not consistent – potentially due to lack of established sleep algorithms for E4 and/or challenges for a wrist-worn device in capturing information on sleep for a SCI population with limited mobility and neurophysiological challenges. 3. EMA is a relatively intense data collection procedure that can be burdensome on the participant. 4. Potential for individuals to change behaviour when they are being monitored.
Kratz et al. 2017a [36] Kratz et al. 2017b [37] Kim et al. 2020c [38]	A) To examine whether pain acceptance moderates the momentary associations of pain intensity with pain interference and physical activity in people with chronic pain and SCI. B) To examine study compliance, protocol acceptability, and reactivity of intensive data collection methods in adults with chronic pain and SCI. C) To examine the moderating effect of within- and between-person pain acceptance on associations between pain and physical and psychosocial functioning.	A) Prospective observational cohort study. B) Secondary analysis of prospective observational cohort study. C) Secondary analysis of prospective observational cohort study.	A) United States (June 2014–January 2016)	*n* = 128 individuals with chronic pain and SCI aged ≥18 years.	Interval and event. 1. Five times throughout the day for a week (upon waking, 11 am, 3 pm, 7 pm, and bedtime)	1. A wrist-worn accelerometer called the (PRO-Diary). 2. EOD electronic diaries (online collection site).	1. Average physical activity per minute. 2. Brief Pain Inventory on 10-point scale. 3. Chronic Pain Coping Inventory-42 (CPCI). 4. Chronic Pain Acceptance 8 (CPAQ8). 5. Pain Catastrophizing using the catastrophizing subscale from the Coping Strategies Questionnaire. 6. Spinal Cord Injury – Quality of Life (SCI-QOL).	1. Depressive symptoms using the PHQ-9.	1. Ability to Participate in Social Roles and Activities using 6 items from the SCI-QOL v1. 2. Positive Affect and Well-Being using the SCI-QOL v1.0. 3. Mobility assessed using the Basic Mobility items in the Spinal Cord Injury-Function Index.	A) **Facilitators** 1. Dynamically demonstrates ecological validity. 2. Combination of objective and subjective measures reduced problems related to overlapping method variance. B) **Facilitators** 1. EMA ratings were completed on a wrist-worn monitor that was constantly accessible. 2. Audible cue prompted the participant to enter data which increased midday compliance. 3. Compliance stayed consistent over the 7-day period. 4. Technologies were not cumbersome and allowed for easy completion of assessment. **Barriers** 1. User error or internet connection difficulties interfered with completion of assessments. 2. It was more difficult for subjects to enter ratings during busy wake and bed-time routines, which, for people with SCI, often involve lengthy and assisted self-care routines (e.g. bowel and bladder care). C) **Facilitators** 1. Daily assessments captured daily fluctuations in pain-related variables. 2. Ecological validity is demonstrated. **Barriers** 1. Subjective daily measures may be inaccurate. 2. Measuring pain-related variables at the end of the day, prevented consideration of within-day variability.
Todd et al. 2018a [39] Todd et al. 2019b [40]	A) To utilize EMA to measure intra-individual diurnal variations in neuropathic pain and effect on exercise and non-exercise days. B) To describe strategies necessary to adapt EMA to measure neuropathic pain in adults with SCI, and explore participant perceptions of using EMA to measure pain sensations.	A) Prospective cohort study. B) Secondary analysis of prospective cohort study.	Canada (Unspecified date)	*n* = 6 physically active men with SCI greater than 1 year post-injury with low neuropathic pain. Participants aged 27–50 years	Random. A total of 6 prompts per day, over 6 days.	1. EMA surveys were administered via mobile EMA mobile application (Ilumivu, Inc). 2. The application utilised push notifications prompting the participant to open the application. 3. Fitbit Surge wrist-worn heart rate (HR) monitors were worn by participants to collect HR data.	1. Modified Neuropathic Pain Scale rated using 10-item Likert scale.	1. 11-point, single item Feeling Scale (FS) measured affect. 2. Felt Arousal Scale (FAS) measured arousal.	1. Heart rate captured using the Fitbit surge HR monitor.	A) **Facilitators** 1. Little missing data and no participant dropout. 2. The measurement schedule was not cumbersome, and therefore minimized the probability of reduced data quality and quantity. **Barriers** 1. Pain may have been exacerbated due to repeatedly asking participants to think about their pain levels. B) **Facilitators** 1. Participants provided positive responses regarding the practicality and usefulness of EMA to accurately capture their neuropathic pain experience. 2. Ecological validity was demonstrated. 3. Participants appreciated minimal morning/ night-time interference. 4. Random EMA allowed for the dynamic phenomenon of neuropathic pain to be captured, while minimizing daily interference to participants. 5. The 6-day protocol could partially explain participants’ support for six assessments per day as the protocol length may have reduced the overall burden experienced by participants. 6. Easy user interface allowed for participants’ positive observations related to the “quick” nature of assessments. 7. Use of notifications to prompt participants was viewed as useful. **Barriers** 1. Participants reported that receiving multiple EMA prompts negatively influenced their neuropathic pain perception.
**Traumatic injury, including head injury**										
Gonzalez-Borato et al. 2021 [41]	To evaluate Psixport’s ability to gather real-time information about injured athletes’ psychological responses during the rehabilitation, to test the users’ perceived usability of Psixport, and to compare the reliability and differences between real-time data gathered with Psixport and the data gathered through the one-time retrospective method.	Prospective cohort feasibility study	Spain (unspecified date)	*n* = 28 severely injured athletes that require surgery and have a rehabilitation prescription therapist after surgery. Participants aged≥18 years.	Event. Daily after completing rehab session for 15 days.	1. Questionnaires completed on Psixport app via mobile phones.	1. Two questions from the Universal Pain Assessment Tool were included in Wong-Baker’s Faces Pain Rating Scale format	1. An adaptation of the picture-oriented Self-Assessment Manikin to assess emotional valence and arousal. 2. Behavioural Responses captured using Sports Injury Rehabilitation Adherence Scale (SIRAS).	1. Psychological Responses to Sports Injury Inventory (PRSII) assessed cognitive appraisals regarding injuries on 6 dimensions: devastation, dispirited, re-organisation, feeling cheated, restlessness, and isolation.	**Facilitators** 1. Users found interface app interface simple and easy. 2. Relatively short questionnaire length allowed for commitment by participants. 3. Ecological validity and validity of responses maximised. 4. Users were able to monitor own progress and rehabilitation. **Barriers** 1. Compliance reduced with each passing day.
Pacella et al. 2018 [42]	To examine changes in post- concussive symptoms (PCS) over the acute post-injury recovery period, focusing on how daily PCSs differ between mTBI and other injury types.	Prospective cohort study.	United States (April 2013 – March 2014)	*n* = 108 adults with traumatic injury aged ≥18 years.	Interval. 3 times per day (9 am, 1 pm, 5 pm) for 14 days. Different symptoms (i.e. somatic, cognitive, emotional) were assessed at each interval.	1. Assessments sent and completed via text message.	1. Somatic measures (i.e. headaches) using a 5-point scale of headache intensity.	1. Cognitive difficulty measures (i.e. concentration) using a 5-point scale. 2. Emotional anxiety and irritability using a 5-point scale.	N/A	**Facilitators** 1. Simple technology and using text-message allowed for easy completion for participants. **Barriers** 1. There may be reporting and recall biases associated with the EMA pattern.
Pacella et al. 2018 [43]	To apply ESM via daily text messaging to monitor and detect relationships among psychosocial factors and post-injury pain across the first 14 days after emergency department (ED) discharge.	Prospective observational cohort study.	United States (January 2016 – May 2017)	*n* = 75 adults with a trauma-related injury aged 18–60 years.	Interval. Five assessments at 5 pm for 14 days.	1. Five assessments sent and completed via text message.	1. Pain rated on a 1–10 Likert scale.	1. Hyperarousal rated on a 1–7 Likert scale.	1. Social support rated on a 1–7 Likert scale. 2. Intrusions rated on a 1–7 Likert scale. 3. Avoidance rated on a 1–7 Likert scale.	**Facilitators** 1. ED patients were receptive to the manner of which assessments were conducted. 2. High compliance rate despite relatively high volume of text messages. **Barriers** 1. Validity was limited as assessment was completed once per day.
Price et al. 2014 [44]	To determine the proportion of trauma patients that would consent to receiving daily text messages assessing mental health, determine response rates to daily text messages among trauma patients, identify predictors of higher rates of responding, assess patient satisfaction, and determine provider burden.	Prospective cohort pilot study	United States (Unspecified date)	*n* = 29 individuals with a traumatic injury. Mean age of 37.1 years.	Interval. A daily assessment for 15 days. One and three month follow-up.	1. Assessments sent and completed via text message.	1. Pain rated using a 1–10 Likert scale	1. Hypervigilance rating using a 1–7 Likert scale. 2. Avoidance rated using a 1–7 Likert scale 3. Re-experiencing rated using a 1–7 Likert scale.	1. Social support rated using a 1–7 Likert scale	**Facilitators** 1. Text messages are an efficient method of implementing a “watchful waiting” program after a traumatic event. **Barriers** 1. Technical difficulties reported as the primary reason for non-response.
Price et al. 2017 [45]	To evaluate the use of a mobile phone application to collect symptom data during the acute post-trauma period.	Prospective cohort study.	United States (Unspecified date)	*n* = 23 individuals with traumatic injury. Mean age of 27.6 years.	Random. Daily assessments between 7 am-8 pm for 30 days. One and three month follow-up.	1. Conducted via mobile application, Metricwire (Waterloo, ON). 2. Follow-up interviews were conducted via telephone.	1. Pain rated using a 1–10 Likert scale	1. Arousal rated using a 1–7 Likert scale. 2. Avoidance rated using a 1–7 Likert scale 3. Re-experiencing rated using a 1–7 Likert scale. 4. Free-text response to what concerned them most that day.	1. Sleep rated using a 1–7 Likert scale.	Facilitators 1. The use of mobile devices to monitor symptoms presents a low-burden and low-cost method with substantial reach to learn about recovery. 2. Most participants felt that 1 survey per day was appropriate. 3. Participants stated that the notifications to complete tasks were helpful. 4. Ease of use of the interface, familiarity with the mobile device, and brevity of the survey allowed for easy completion of survey. 5. Using an individual’s personal device significantly reduced the cost associated with conducting studies or delivering intervention. Barriers 1. Participants may have preferred an opportunity to respond more frequently but did not feel an overwhelming obligation to do so. 2. Participants found the inclusion of the same questions in each survey repetitive, which may have diminished their willingness to respond to subsequent assessments. 3. Participants requested personalized questions and personalized feedback for a better experience. 4. Responding to assessments via a mobile device may be burdensome to the patient and might result in noncompliance.
Price et al. 2018 [46]	To evaluate the acceptability of administering PTSD symptom assessments via a mobile application throughout the acute post-trauma period.	Prospective cohort study	United States (Unspecified date)	*n* = 90 individuals with traumatic injury (*n* = 1 participant was injured following a physical assault). Mean age of 35.1 years.	Random. Daily assessments between 7 am-8 pm for 30 days. One and three month follow-up.	1. Conducted via mobile application, Metricwire (Waterloo, ON).	1. Pain rated on a 1–10 Likert scale.	1. Post- Traumatic Stress Disorder (PTSD) checklist-5 (PCL-5) to assess PTSD symptoms (8-items). 2. Free-text response to what concerned them most that day.	1. Sleep assessment using PCL-5.	**Facilitators** 1. Assessments were perceived as moderately helpful and minimally burdensome. 2. Demonstrates possibility to obtain free response and Likert scale response via EMA. **Barriers** 1. A subset of participants may not complete daily assessments due to technical difficulties or a lack of interest. 2. Variability in response time. Many took advantage of the 14-h response window.

*N/A* Not applicable

**Fig. 2:**
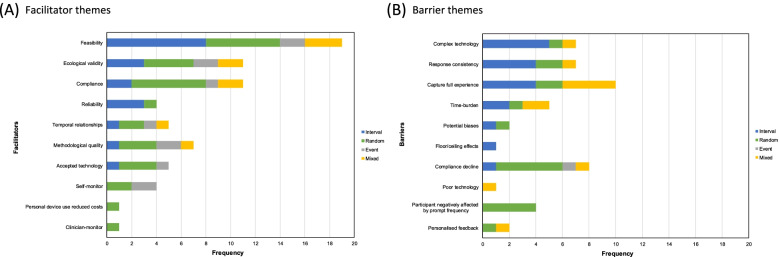
Key facilitator (**A**) and barrier (**B**) themes identified for ecological momentary assessment (EMA) by EMA type^1^ ^1^Multiple themes could be identified for each article.

There were ten key facilitator themes and ten key barrier themes identified in studies using EMA to examine health outcomes post-injury (Table 2). Where facilitators were identified, feasibility, ecological validity, and compliance were the most common themes identified for each EMA type (Fig. 2a). Complex technology, response consistency, and the ability to capture a participant’s full experience were the most common barrier themes identified for interval EMAs. Compliance decline and the potential for participants to be negatively affected by prompt frequency were the most common barrier themes identified for random EMAs (Fig. 2b). Quality assessment measures for articles varied and few studies (24.0%; *n* = 6) received all ‘Yes’ ratings (Tables 3 and 4).

**Table 2:** Identified ecological momentary assessment facilitator and barrier themes

Theme	Examples	Authors and EMA type
**Facilitators**		
Accepted technology	Ease of use of the interface, familiarity with the mobile device, and brevity of the survey allowed for easy completion of survey. Does not take very long for participants to habituate to a monitoring device (i.e. E4).	Price et al. [45] - random Carlozzi et al. [47] - interval
Clinician-monitor	Ability for results to be shared with the treating clinician.	Forster et al. [20] - random
Compliance	Relatively short questionnaire length allowed for commitment by participants. A schedule feature resulted in great compliance and satisfaction.	Gonzalez-Borato et al. [41] - event Juengst et al. [22] - interval
Ecological validity	Higher frequency repeated symptom assessment in a natural environment over a short period could provide a more valid measure of emotional symptoms and a better indicator of clinically meaningful change at the individual level. Data collection method had little influence on mood and feelings promoting ecological validity.	Juengst et al. [23] - interval Lenaert et al. [24] - random
Feasibility	Simple technology and using text-message allowed for easy completion for participants. Text messages are an efficient method of implementing a “watchful waiting” program after a traumatic event.	Parcella et al. [42] - interval Price et al. [44] - interval
Methodological quality	Combination of objective and subjective measures reduced problems related to overlapping method variance. Mobile ecological momentary assessments (mEMA) better predicted recovery time than Post-Concussion Symptom Scale (PCSS).	Kratz et al. [36] – interval and event Sufrinko et al. [30] - random
Personal device use reduced costs	Using an individual’s personal device significantly reduced the cost associated with conducting studies or delivering intervention.	Price et al. [45] - random
Reliability	Use of EMA may reduce misidentification of individuals with clinically significant symptoms. Data collection minimises recall bias.	Juengst et al. [23] - interval Carlozzi et al. [47] - interval
Self-monitor	Patients able to assess and monitor their own symptoms. Users were able to monitor own progress and rehabilitation.	Forster et al. [20] - random Gonzalez-Borato et al. [41] - event
Temporal relationships	Ability to examine temporal relationships among symptoms. mEMA data across recovery better predicted recovery duration compared with PCSS score at any clinic visit, but illustrated symptom patterns that may further inform clinical profiles and guide treatment recommendations.	Carlozzi et al. [47] - interval Sufrinko et al. [30] - random
**Barriers**		
Capture full experience	Pain in spinal cord injury (SCI) is multifaceted, and thus ratings of pain intensity do not capture the full breadth of the pain experience in individuals with SCI. Single daily assessments may not be a valid representation of symptoms/ progression.	Carlozzi et al. [34] - interval Juengst et al. [23] - interval
Complex technology	EMA requires more sophisticated analytical hardware for monitoring and data capture. User error or internet connection difficulties interfered with completion of assessments.	Carlozzi et al. [34] - interval Kratz et al. [37] – interval and event
Compliance decline	Compliance decreased with every passing testing day. Participants were less likely to respond as days since injury increased.	Forster et al. [20] - random Sufrinko et al. [30] - random
Response consistency	Difficult to gauge how reliable and realistic the patients answers were. Method of self-reporting may contribute to variability.	Forster et al. [20] - random Juengst et al. [23] - interval
Floor/ceiling effects	The presence of a floor effect for EMA pain interference presented an analytical challenge in our data.	Carlozzi et al. [34] - interval
Potential biases	Potential for individuals to change behaviour when they are being monitored. The use of EMA might also be limited by other psychological factors such as social desirability and patients’ individual differences.	Carlozzi et al. [47] - interval Forster et al. [20] - random
Personalised feedback	Participants requested personalized questions and personalized feedback for a better experience. Participants indicated that they did not believe the data they were providing were doing any good because they could not see any effect on their treatment. On a few occasions participants temporarily ceased participating in EMA data collection after researchers and clinicians failed to respond to a stress or crisis event recorded in EMA data.	Price et al. [45] - random Smith et al. [28] – interval, event, and random
Participant negatively affected by prompt frequency	Pain may have been exacerbated due to repeatedly asking participants to think about their pain levels. Participant mood and emotion may have been influenced by high frequency of prompts.	Todd et al. [39] - random Forster et al. [20] - random
Poor technology	The PDAs were viewed as being clunky and out of date compared with smart phones.	Smith et al. [28] interval, event, and random
Time-burden	EMA is a relatively intense data collection procedure that can be burdensome on the participant. It was more difficult for subjects to enter ratings during busy wake and bed-time routines, which, for people with SCI, often involve lengthy and assisted self-care routines (e.g. bowel and bladder care).	Carlozzi et al. [47] - interval Kratz et al. [37] – interval and event

**Table 3:** Quality assessment criteria of each article using CASP Cohort Checklist^a^

Authors	Q1	Q2	Q3	Q4	Q5a	Q5b	Q6a	Q6b	Q7	Q8	Q9	Q10	Q11	Q12
**Traumatic brain injury, concussion, and acquired brain injury**														
Albanese et al. [18]	Y	Y	Y	Y	Y	Y	C	Y	Greater distress intolerance predicted a poorer ability to volitionally suppress intrusions during the monitoring period.	Y	Y	Y	Y	Y
Forster et al. [20]	Y	Y	Y	Y	N	Y	Y	Y	Patients’ mean compliance rate for EMA was 71.6%. Across all variables, a mean of 55.1% variability in responses. No correlation between patients’ compliance or mean fluctuation.	Y	Y	Y	Y	Y
Juengst et al. [22]	Y	Y	Y	Y	Y	Y	Y	Y	Participants correctly completed 73.4% of all scheduled assessments	Y	Y	Y	Y	Y
Juengst et al. [23]	Y	Y	Y	Y	N	Y	Y	Y	Significant temporal within-person variability occurred for all measures.	N	Y	Y	Y	Y
Lenaert et al. [24]	Y	Y	Y	Y	N	Y	Y	Y	Demonstrated feasibility with a 71% response rate and a 99% completion rate. There were no dropouts and method indicated as user-friendly.	N	Y	Y	Y	Y
Rabinowitz et al. [26]	Y	Y	Y	Y	Y	Y	Y	Y	Network modelling revealed marked heterogeneity across participants in terms of acute concussion symptoms.	Y	Y	Y	C	Y
Rabinowitz et al. [27]	Y	Y	Y	Y	Y	Y	N	Y	EMA response rate was positively correlated with integrity of episodic memory and education. Activities associated with positive or negative affect were able to be characterised.	N	Y	Y	Y	Y
Smith et al. [28]	Y	Y	Y	Y	N	N	Y	Y	mHealth technologies are feasible adjuncts to traditional medical treatment in the Veteran population.	N	Y	Y	Y	Y
Sufrinko et al. [30]	Y	Y	Y	Y	Y	Y	N	Y	Post-concussion symptoms able to be measured using mobile EMA and symptoms captured able to be used to determine associations with recovery.	Y	Y	Y	Y	Y
Trbovich et al. [31]	Y	Y	Y	Y	Y	Y	C	Y	Sleep efficiency and total sleep time were negatively associated with next day concussion symptoms.	Y	Y	Y	Y	Y
Wiebe et al. [32]	Y	Y	Y	Y	N	N	Y	Y	EMA feasible. Concussion symptoms decreased as the 2-week follow-up period progressed.	Y	Y	Y	C	Y
Worthen-Chaudhari et al. [33]	Y	Y	Y	Y	N	Y	Y	Y	Mobile apps using social gaming may promote health management in teens with unresolved concussion symptoms.	Y	Y	Y	C	Y
**Spinal cord injury**														
Carlozzi et al. [34]	Y	Y	Y	Y	Y	Y	Y	Y	Identified minimum of number of End of Day (EOD) and Ecological Momentary Assessments (EMAs) needed to achieve different levels of reliability (“adequate” > 0.70, “good” > 0.80 and excellent > 0.90).	N	Y	Y	Y	Y
Carlozzi et al. [35]	Y	Y	Y	Y	Y	Y	Y	Y	Fluctuations in sleep quality were significantly associated with ratings of Health-related Quality of Life (HRQOL).	Y	Y	Y	Y	Y
Kratz et al. [36]	Y	Y	Y	Y	Y	Y	Y	Y	Pain acceptance significantly moderated the momentary association between pain intensity and pain interference; those with higher pain acceptance experienced a blunted increase in interference when pain was high.	N	Y	Y	Y	Y
Kratz et al. [37]	Y	Y	Y	Y	Y	Y	Y	Y	Participant compliance was related to time of day/ presence of audible prompts, mobility aid use, race, and baseline levels of pain and pain interference, with more missing data at wake and bedtimes/ no prompts, and for those who used hand-held mobility devices, identified as African American, and/or reported higher baseline pain and pain interference.	Y	Y	Y	Y	Y
Kim et al. [38]	Y	Y	Y	Y	Y	Y	Y	Y	Bivariate correlations indicated moderate to large between-person linear associations between pain acceptance, intensity, and catastrophizing.	N	Y	Y	Y	Y
Todd et al. [39]	Y	Y	Y	Y	Y	Y	Y	Y	Participants experienced a significant decrease in neuropathic pain following completion of at least one bout of exercise.	Y	Y	Y	Y	Y
Todd et al. [40]	Y	Y	Y	Y	N	N	Y	Y	Participants reported that EMA protocol was unobtrusive to their daily routines, and effectively captured their neuropathic pain sensations.	N	Y	Y	Y	Y
**Traumatic injury, including head injury**														
Gonzalez-Borato et al. [41]	Y	Y	Y	Y	N	N	N	Y	Psixport can gather information about injured athletes’ cognitive appraisals, emotional responses, behaviours, and pain perceptions. EMA more accurate than retrospective reports.	Y	Y	Y	Y	Y
Pacella et al. [42]	Y	Y	Y	Y	Y	Y	Y	Y	Greater odds of headache and concentration difficulties on day 1 post-injury among the head injured and mild Traumatic Brain Injury (mTBI) groups vs non-head injured trauma controls.	Y	Y	Y	Y	Y
Pacella et al. [43]	Y	Y	Y	Y	Y	Y	Y	Y	Pain scores decreased over time, and daily fluctuations of hyperarousal were associated with daily fluctuations in reported pain level within each person.	Y	Y	Y	Y	Y
Price et al. [44]	Y	Y	Y	Y	Y	Y	N	Y	Response rates were correlated with PTSD symptoms at baseline but not at other times.	N	Y	Y	Y	Y
Price et al. [45]	Y	Y	Y	Y	Y	N	N	Y	Responses rates were uncorrelated with PTSD symptoms or depression symptoms at 1-and 3-month post-injury.	N	Y	Y	Y	Y
Price et al. [46]	Y	Y	Y	Y	N	N	N	Y	Response rate was 61.1%. Participants reported that the daily assessments were not bothersome and were moderately helpful.	N	Y	Y	Y	Y

*Y* Yes, *N* No *C* Can’t tell, *N/A* Not applicable

^a^*CASP Cohort Appraisal Checklist questions:*

1. Did the study address a clearly focussed issue?

2. Was the cohort recruited in an acceptable way?

3. Was the exposure accurately measured to minimise bias?

4. Was the outcome accurately measured to minimise bias?

5a. Have the authors identified all important confounding factors?

5b. Have they taken into account of the confounding factors in the design and/or analysis?

6a. Was the follow up of the subjects complete enough?

6b. Was the follow up of subjects long enough?

7. What are the result of this study?

8. How precise are the results?

9. Do you believe the results?

10. Can the results be applied to the local population?

11. Do the results of this study fit with other available evidence?

12. Does the study have implications for practice?

**Table 4:** Quality assessment criteria of each article using CASP RCT Checklist^a^

Authors	Q1	Q2	Q3	Q4	Q5	Q6	Q7	Q8	Q9	Q10	Q11
**Traumatic brain injury, concussion, and acquired brain injury**											
Betthauser et al. [19]	Y	Y	Y	N	Y	Y	Y	Y	Y	Y	C
Hart et al. [21]	Y	Y	C	N	Y	Y	Y	N	Y	Y	Y
Pavliscsak et al. [25]	Y	Y	Y	N	Y	Y	Y	C	Y	Y	Y
Suffoletto et al. [29]	Y	Y	Y	Y	N	Y	Y	C	Y	Y	Y

*Y* Yes, *N* No, *C* Can’t tell, *N/A* Not applicable

^a^*CASP RCT Appraisal Checklist questions:*

1. Did the trial address a clearly focussed issue?

2. Was the assignment of patients to treatments randomised?

3. Were all of the patients who entered the trial properly accounted for at its conclusion?

4. Were the patients, health workers and study personnel ‘blind’ to treatment?

5. Were the groups similar at the start of the trial?

6. Aside from the experimental intervention, were the groups treated equally?

7. How large was the treatment effect?

8. How precise was the estimate of the treatment effect?

9. Can the results be applied to the local population?

10. Were all clinically important outcomes considered?

11. Are the benefits worth the harms and costs?

## Discussion

This rapid review identified 25 unique studies where EMAs were used to monitor symptoms and QoL after unintentional traumatic injury. Obtaining this sort of experiential information post-injury can assist in identifying temporal changes in clinically-relevant symptoms and health states, in making decisions regarding further treatment options, in allocating health service and resource requirements, and has the ability to identify any unmet health needs [48].

With mobile technology advances, the use of EMA is likely to become more commonplace to conduct follow-up studies for injured individuals in real-time and in real-world settings. This review has identified that each type of EMA (i.e. interval, random, and event) has demonstrated feasibility to monitor post-injury recovery, commonly for individuals who sustained brain or spinal cord injuries. Individuals who sustain either brain injuries or a spinal cord injury can have a long-term recovery and adjustment period, as individuals and their families adjust to living with the consequences of these injuries and their associated symptoms and related health issues [49–51]. It is possible that EMA could facilitate the long-term monitoring of the recovery of traumatically injured populations.

In general, compliance with data collection in prospective studies that have used EMAs has been reported as high [52], and that EMAs can reduce recall bias through using real-time data collection [23, 34, 47]. While participant responses using EMAs are considered to be more reliable than retrospective studies [22, 31, 48], several study authors did identify as a potential barrier the reliability of information from participants obtained using EMAs [20, 23, 27, 37, 42, 46, 47]. For some participant information, such as health service use (e.g. primary care, emergency department visit or hospital admission), there would be the potential to cross-check information provided by the participant with information from other sources, such as through record linkage of self-reported data collected using EMA with administrative health records [53].

Five studies [30, 33, 38, 40, 47] identified that EMAs facilitated the examination of temporal relationships, such as health symptoms like pain or PTSD and time since injury event. Examining temporal relationships can assist in providing information on factors associated with participant well-being and clinical improvement or where further treatment options could be considered. In addition, capturing information on the use of primary care and allied health services over time for injured individuals could also provide insight into the frequency of use of these services, along with details of treatment or rehabilitative activities undertaken.

For the post-injury cohorts in this review, only three studies collected biometric data using EMA, with heart rate [39, 40] and sleep and movement activity [31] recorded. Biometric data collection measures can be felt by some participants to be intrusive [54], but may become more frequently used over time. Motion-sensor apps have been incorporated into smart phones and it is possible that the ability to unobtrusively capture some participant activities, like walking, balance, and physical activity participation will grow over time [55].

Conducting follow-up studies of injured individuals post-discharge is not without challenges, such as participant retention [19] and compliance with data collection protocols [56]. EMA studies have generally shown good retention [35, 42] and participant compliance with data collection prompts [52]. However, this review has identified several potential barriers to EMA use, including the acknowledged behaviour-altering effects associated with social desirability bias and the Hawthorne Effect [20, 47]. Six studies queried whether it was possible that the full experience of an injured individual was being captured using EMAs [23, 30, 34, 37, 43, 46]. By limiting the number of follow-up questions that participants are asked to reduce time-burden, it is possible that key aspects of a participant’s post-injury experiences are not being captured. Including a free-text option for participants to record any additional information they would like to provide would allow participants the ability to provide pertinent information of their own choosing and could allow a more complete picture of participant experience to be obtained [57].

This review identified that studies that use EMAs should have the functionality to provide feedback to participants regarding the EMA data they have provided to enable each participant to monitor their own progress post-injury, such as progress with their rehabilitation activities [20, 24, 41, 58]. There was a decline in participant compliance with data collection prompts identified in some studies [20, 25, 28, 30, 41, 46]. However, potential mechanisms to retain participant compliance over time could include gamification of some aspects of data collection [58] and providing participant feedback regarding their progress in real-time.

In general, health symptoms tend to be reported as more intense and longer lasting using EMAs [5]. In four studies, the authors acknowledged that participants appeared to be negatively affected by prompt frequency [20, 30, 39, 40], demonstrating the need to have in place appropriate risk management practices [59] to reduce any adverse impact on study participants. Risk management practices could include the use of clinical thresholds for participant responses (including for pain scores or psychological health measures) to trigger immediate follow-up of the participant from a health professional. There is the potential that some health outcome measures may need to be adapted for use with EMA to reduce the potential for participant re-experiencing [46] of the injurious event or feelings around the injurious event, such as might occur through the collection of information on PTSD symptoms.

### Strengths and limitations

The strengths of this rapid review were that it used a comprehensive search strategy involving multiple databases, the review followed the PRISMA guideline, a specialist university librarian assisted with the development of the key search terms, and multiple reviewers were involved in the data extraction phase with high interrater reliability. Any clarifications or disagreements were discussed between reviewers and consensus was obtained. However, there were limitations. Only unintentional injuries were considered, therefore articles that used EMA to examine health outcomes following self-harm were excluded and these may have contributed additional insights. Articles published in non-English languages were excluded, which may result in language-bias. The identification of facilitator and barriers was reliant on the reporting of these factors in articles by study authors. The relationship between key facilitator and barrier themes is not known and Fig. 2 results are likely influenced by the frequency that different EMA types were used by different studies.

### Future directions

There are several further opportunities for research using EMAs. As meanings can differ for participant responses using EMAs in different countries, further exploration and establishment of response norms for different types of injuries would be advantageous [60]. There is also potential for other factors, such as type of setting or presence of peers, to influence participant data recording practices, therefore the generation of normative samples (e.g. using non-injured participants) could assist to tease out the influence of some of these factors [61]. Normative samples may also provide information on exposure and risk factors for injury [59], along with the potential for exposure-time estimates for different activities (e.g. worker hazard exposure, time spent playing or training for different sports) [9, 62, 63] using activity sensor technology. There is also the potential for EMAs to be used to record information regarding the perceptions and experience of family members [64] of an injured individual. More broadly, health systems could incorporate long-term symptom and QoL measures using EMAs as part of routine patient follow-up, with any symptom reports outside a normative range being flagged for clinical follow-up and assessment [23].

## Conclusion

This review summaries the literature on the use of EMAs to capture symptoms, health states, behaviours, QoL, and activities post-injury. It highlighted the usefulness of EMA to capture ecologically valid participant responses of their experiences post-injury and has identified common facilitators and barriers regarding the use of EMAs. EMAs have the potential to assist in routine clinical follow-up of health outcomes of patients post-injury and their use should be further explored.

## Supplementary Information

**Additional file 1.**

## Data Availability

All data generated or analysed during this study are included in this published article.

## Abbreviations

ABI: Acquired brain injury
ADL: Activity of daily living
ED: Emergency department
EMA: Ecological momentary assessment
EOD: End of day
ESM: Experience sampling method
HRQoL: Health-related quality of life
mTBI: Mild traumatic brain injury
N: not applicable
PCS: Post-concussive symptoms
PTSD: Post-traumatic stress disorder
QoL: Quality of life
RCT: Randomised control trial
SCI: Spinal cord injury
TBI: Traumatic brain injury
