# Supplementary material for: Synthesis of evidence on the use of ecological momentary assessments to monitor health outcomes after traumatic injury: rapid systematic review
Source: BMC Med Res Methodol. 2022 Apr 22;22:119. doi: 10.1186/s12874-022-01586-w (PMC9027879; doi:10.1186/s12874-022-01586-w)
Supplement: Supplementary file 1 — Additional file 1. [file 12874_2022_1586_MOESM1_ESM.docx]

**Appendix 1: Full search strategy**

### **Medline**

(ecological momentary assessment OR Ecological Momentary Assessment OR momentary assessment* OR EMA OR experience sampling OR ambulatory assessment* OR event sampling) AND (injury OR wounds and injuries OR trauma* or injur* or wound*)

### **PsycInfo**

(ecological momentary assessment OR Ecological Momentary Assessment OR momentary assessment* OR EMA OR experience sampling OR ambulatory assessment* OR event sampling) AND (injury OR brain injuries OR injuries OR head injuries OR electrical injuries OR spinal cord injuries OR wound OR trauma)

### **Scopus**

(TITLE-ABS-KEY ( "ecological momentary assessment*" OR "momentary assessment*" OR "ema" OR "experience sampling" OR "ambulatory assessment*" OR "event sampling" ) AND TITLE-ABS-KEY ( injur* OR trauma* OR wound*))

### **Web of Science**

(TOPIC ( "ecological momentary assessment*" OR "momentary assessment*" OR "ema" OR "experience sampling" OR "ambulatory assessment*" OR "event sampling" ) AND TOPIC ( injur* OR trauma* OR wound* OR "physical injur*"))

### **Embase**

(ecological momentary assessment OR Ecological Momentary Assessment OR momentary assessment* OR EMA OR experience sampling OR ambulatory assessment* OR event sampling) AND (injury OR wound OR trauma)

### **Academic Search Premier, CINAHL, Psychology and Behavioural Sciences Collection, SPORTDiscus, and CINAHL**

(ecological momentary assessment OR Ecological Momentary Assessment OR momentary assessment* OR EMA OR experience sampling OR ambulatory assessment* OR event sampling) AND (injur* OR trauma* OR accident* OR wound* OR lesion* OR bruise* OR abrasion* OR harm)
